# NT-ProBNP levels are moderately increased in acute high-altitude pulmonary edema

**DOI:** 10.3892/etm.2013.976

**Published:** 2013-02-26

**Authors:** MINGDONG GAO, RUIMIN WANG, ZEPEI JIAYONG, YIN LIU, GENYI SUN

**Affiliations:** 1 Department of Cardiology, Tianjin Chest Hospital, Tianjin 30051;; 2 Department of Clinical Laboratory, Affiliated Hospital of Logistics University of Chinese People’s Armed Police Forces, Tianjin 300162;; 3 Changdu District People’s Hospital, Tibet 854000, P.R. China

**Keywords:** BNP, high-altitude pulmonary edema, altitude sickness

## Abstract

The aim of the present study was to investigate the effect of B-type natriuretic peptides (BNPs) in acute high-altitude pulmonary edema (HAPE). The study enrolled 46 subjects from lowland Han, including 33 individuals who had acutely ascended to a high altitude (21 individuals with HAPE as the case group and 12 individuals without HAPE as the high-altitude control group) and 13 healthy normal residents as the plain control group. The serum concentrations of N-terminal probrain natriuretic peptide (NT-proBNP), erythropoietin (EPO), vascular endothelial growth factor (VEGF) and nitric oxide (NO) were measured. There were significant differences in the serum concentrations of NT-ProBNP, NO, VEGF and EPO among the three groups. The serum concentrations of NT-ProBNP, EPO and VEGF were significantly higher in the HAPE patients and high-altitude control individuals than those of the plain group. No significant differences were identified between the HAPE patients and the high-altitude control group. In contrast to these three parameters, the serum concentrations of NO in the high-altitude control group were significantly higher than those of the HAPE patients and the plain group, while there were no significant differences in the serum concentrations of NO between the HAPE patients and the plain group. Furthermore, serum concentrations of NT-ProBNP and EPO were significantly reduced following treatment in the HAPE patients, however, no significant changes were identified in VEGF or NO concentrations. BNPs are increased in HAPE with severe hypoxia and right ventricular overload, but are decreased subsequent to treatment. BNPs may therefore be a potential biomarker for the diagnosis and prognosis of HAPE.

## Introduction

Acute high-altitude pulmonary edema (HAPE) is a form of non-cardiogenic pulmonary edema with a normal pulmonary capillary wedge pressure. HAPE typically occurs in healthy, young individuals who ascend rapidly to altitudes >2,500 m or in residents who reascend from low altitudes to altitudes >3,000 m and/or engage in vigorous physical activities. Pulmonary edema results from the conjunction of alveolar flooding, induced by exaggerated hypoxic pulmonary hypertension, and impaired alveolar fluid clearance, which are associated with defective pulmonary transepithelial sodium transport (1). Molecular mechanisms that have been reported in HAPE cases include: i) exaggerated sympathetic activation (2), ii) defective synthesis of NO (3) or reduced bioavailability of NO mediated by free-radicals (4), iii) exaggerated endothelin-1 (ET-1) synthesis or reduced ET-1 clearance (5,6) and iv) overexpression of vascular endothelial growth factor (VEGF) (7). Natriuretic peptides have also been demonstrated to be involved in the control of hypoxic pulmonary vasoconstriction (8). However, the B-type natriuretic peptide (BNP) responses in HAPE patients remain largely unknown.

BNPs are released primarily from the ventricular myocardium in response to increased ventricular wall stress. Hypoxia is a direct, potent stimulus for BNP secretion (9). The measurement of BNP has routinely been used as a test for the diagnosis of heart failure. In particular, one study suggested the significance of BNP in pulmonary artery hypertension patients with or without right ventricular dysfunction (10). BNPs play a significant role in body fluid homeostasis and the regulation of blood pressure (11). In addition, BNPs possess antihypertrophic and antifibrotic properties, implying that they have powerful cardioprotective effects (12). In order to investigate the characteristics of BNP in HAPE, NT-proBNP, a cleavage product of the prohormone that is more stable than BNP, was measured in the serum of the HAPE patients and the controls, along with other factors associated with HAPE. The erythropoietin (EPO) level was measured as a marker for the degree of the system response to hypoxia, while NO and VEGF were measured as markers of hypoxia-induced systemic endothelial function.

## Subjects and methods

### Study population

During the spring of 2010, from a total of 33 native Han individuals who had rapidly ascended from low (height <500 m) to high altitudes (height >3,000 m), 21 individuals diagnosed with HAPE and 12 individuals without HAPE were selected for the present study according to the diagnostic criteria of HAPE (13). A diagnosis of HAPE was made clinically based on the symptoms and signs gathered from an ascent to a high altitude and when no clinical evidence was identified of another cause for the hypoxemia. All chest radiographs were carefully examined to either diagnose or exclude HAPE. Chest radiographs were checked again subsequent to clinical recovery. Each diagnosis was established clinically by the same heart physician. A total of 13 age- and gender-matched normal Han individuals were randomly selected as the plain controls. Participants suffering from cardiopulmonary or altitude-related diseases were excluded.

Subsequent to being informed of the study protocol and providing written consent of their participation in the study, the subjects were interviewed and a physical examination was performed. Prior to recovery, all HAPE patients were treated in the Changdu District People’s Hospital, which is located 3,200 m above sea level in the eastern part of Tibet in China.

### Measurement of biochemical parameters

Venous blood was drawn from every subject for biochemical analysis. The serum was separated immediately and stored at −80°C until analysis. The blood samples obtained from the healthy lowland controls were transported to the Changdu laboratory by air to analyze them together with the other specimens. The serum concentrations of NT-proBNP, EPO, NO and VEGF were tested using commercially available kits (Biomedical Gruppe, Vienna, Austria).

### Statistical analysis

The categorical variables are presented as proportions and were compared using the Chi-square tests. Continuous variables are presented as the median and the 25th and 75th percentiles and were compared using non-parametric tests. A Kruskal-Wallis H test and a Mann-Whitney U test were used for comparisons among the HAPE patients and the 2 control groups. A Wilcoxon test was used to compare the results for HAPE patients pre- and post-treatment. P<0.05 was considered to indicate a statistically significant difference. All statistical procedures were performed using the SPSS 13.0 (SPSS, Inc., Chicago, IL, USA) statistical package.

## Results

The basic characteristics of the study subjects are shown in Table I. The clinical characteristics for the HAPE patients, including symptoms, physical signs and the results of the laboratory tests, are shown in Table II.

When the 3 groups were compared there were significant differences in the serum NT-ProBNP, EPO, VEGF and NO concentrations (P=0.010, 0.001, 0.002 and 0.013, respectively). The serum concentrations of NT-ProBNP, EPO and VEGF were significantly higher in the HAPE patients and the high-altitude control individuals than those of the plain controls (P<0.05, Table III). There were no significant differences identified between the HAPE patients and high-altitude control individuals for these three parameters. By contrast, the serum concentrations of NO were significantly higher in the high-altitude control group than those of the HAPE patients and the plain group (P<0.05, Table III). There were no significant differences in the serum concentrations of NO between the HAPE patients and the plain group (Table III).

As a number of the HAPE patients left the hospital early, only 5 patients had any measurements repeated following the resolution of their symptoms. These results are shown in Table IV. Significant reductions in the NT-ProBNP and EPO levels were identified subsequent to treatment (both P= 0.043), but there were no significant changes in the VEGF or NO levels (P=0.500 and 0.686, respectively).

## Discussion

The results of the present study show that patients with HAPE have elevated NT-proBNP levels accompanied by higher concentrations of VEGF and EPO when compared with the plain control group, and lower NO concentrations than those of the plain or high-altitude control groups. However, the elevated NT-proBNP levels were not high enough to diagnose heart failure. This finding is compatible with the original hypothesis of the present study and is similar to the findings of Ge *et al*(14).

A number of travellers visiting Tibet have developed HAPE, particularly those traveling by plane from the lowland region, as they have not become acclimatized to the high altitude. The causative factors identified for HAPE involve catching colds, drinking habits and excess fatigue. In the present study, subjects who were diagnosed with HAPE 5 times during 5 years may be considered as habitual HAPE sufferers. Due to delayed treatments, 4 subjects suffered acute high-altitude cerebral edema (HACE) as a complication. There were also three subjects who presented with hypertension and were consequently administered angiotensin-converting enzyme inhibitor (ACEI). Diuretics or glucocorticoids were also administered to certain patients to eliminate edema.

A previous study on ischemic coronary heart disease patients showed that the serum concentrations of BNP were always increased regardless of whether these patients had normal or impaired left ventricular ejection fractions (15). However, a second study showed that when cultured human ventricular myocytes (AC16 cells), regulated by hypoxia induced factor-1 (HIF-1), were exposed to hypoxia, BNP secretion was elevated following the augmentation of BNP gene expression, with the occurrence of activated HIF-1 identified earlier than the elevation of BNP gene expression (16). This suggests that the hypoxia-induced alterations in the BNP activity were determined at the transcriptional and translational levels (16,17). The hypoxic induction of BNP was also paralleled by the similar expression pattern and protein secretion of VEGF, another HIF-1 target factor (18). Together, these findings suggest that hypoxia directly enhances the synthesis and secretion of BNP in cadiocytes through a HIF-1α-dependent mechanism (9,17). Identical to the findings of the present study, an increased NT-proBNP concentration was accompanied by an increased VEGF and EPO concentration. Ge *et al*(14) observed that patients with chronic mountain sickness (CMS) had higher concentrations of BNP which were proportionate to their higher pulmonary artery pressures. Furthermore, higher concentrations of BNP correlated with higher concentrations of VEGF and ET-1. However, these patients also had lower concentrations of endothelial NO synthase (eNOS).

In the present study, the NT-proBNP levels in the HAPE patients were significantly higher than those of the plain control group, although they were all lower than the diagnostic cut-off value for heart failure (19). Numerous factors are involved in HAPE, including reduced myocardial blood flow reserve (20), right ventricular overload, increased ventricular wall stress, abnormal left ventricular diastolic function (21) and changes to the transmitral inflow pattern, thus implying impaired left ventricular diastolic function (22,23). It is likely that any one of those factors is not sufficient to cause heart failure, which itself may be a triggering agent for BNP secretion in HAPE. In fact, there does not appear to be a reason that there are only differences in assay methodology present to explain the lack of increased NT-proBNP or BNP levels recorded by Toshner *et al*(24) and Feddersen *et al*(25). However, a significant rise in the levels of BNP or NT-proBNP at high-altitude have now been observed in two recent studies (26,27) and, more persuasively, none of the subjects in these studies developed HAPE or HACE.

The major physiological effects of BNP are vasodilatation, natriuresis and inhibitation of the rennin-angiotensin-aldosterone and sympathetic nervous systems (28). BNP aids in avoiding the more severe hypoxic injuries in acute hypoxia and even in avoiding hypertrophy and fibrosis of cardiocytes in chronic hypoxia (12). However, the effects of diuresis and natriuresis do not correlate with BNP levels (25). It is accepted that higher levels of BNP are an indicator of an increased right ventricular load due to pulmonary artery hypertension and that the degree of elevated right heart strain may be estimated by measuring BNP or NT-proBNP concentrations (29). It may also be supposed that BNP acts as an endogenous pulmonary vasodilator, which modulates the hypoxic response (development of pulmonary artery hypertension or cardiac hypertrophy) and promotes acclimatization. However, the present study has not confirmed the role that BNP plays in the development of HAPE. The correlation of HAPE severity and BNP remains to be further investigated.

All the patients in the present study were treated in the high-altitude hospital prior to recovery, therefore there was no significant decrease in the serum levels of VEGF and no significant increase in NO subsequent to the treatment. NT-proBNP and EPO levels were significantly decreased following treatment. Together with EPO, BNP may promote resistance to hypoxia and improve accommodation through its powerful cardioprotective effects (12,30). The NO levels in the present study were not significantly increased in HAPE patients prior and subsequent to treatment, but a significant increase was observed in non-susceptible individuals. This provides important evidence that the shortage or reduced pulmonary bioavailability of NO may play a partial role in HAPE pathogeny (3,4).

The present study confirmed that NT-proBNP serum levels were significantly elevated in HAPE and that they decreased subsequent to treatment, accompanied by changes in other vasoactive substances. A decreased NT-proBNP level may be a biomarker for the recovery of HAPE subsequent to treatment. However, due to the small number of subjects in the present study, these findings require corroboration in larger groups. This is likely to lead to an improved understanding of the roles and mechanisms of BNP in HAPE.

## Figures and Tables

**Table I:** Basic characteristics of the study subjects.

Characteristics	HAPE (n=21)	High-altitude controls (n=12)	Plain controls (n=12)
Age (years), mean ± SD	31.24±11.47	36.54±10.28	35.38±10.63
Gender, male (%)	18 (85.7)	10 (83.3)	9 (75)
BMI (kg/m^2^), mean ± SD	23.85±1.21	24.33±1.29	24.07±1.19
Hypertension, n (%)	4 (19.0)	3 (25)	0 (0)

BMI, body mass index; HAPE, high-altitude pumonary edema.

**Table II:** Clinical characteristics of the HAPE patients (n=21).

Factors and characteristics	Ratio (%)
Symptoms and physical examination	
Moist rales in lung	21 (100)
Sinus tachycardia	14 (66.7)
Ischemic change of ST segment and/or T wave of ECG	10 (47.6)
Fever	2 (9.5)
Heart murmur	3 (14.3)
Supine position difficult	4 (19)
Bloody sputum	10 (47.6)
HACE	4 (19)
Increased WBC	8 (38.1)
Pulmonary edema on chest X ray	
Unilateral lung	2 (9.5)
Bilateral lung	15 (71.4)
Interstitial	4 (19.1)
SO_2_	
<70%	2 (9.5)
70–90%	16 (76)
>90%	3 (14.5)
Instance of illness	
First time	16 (76.2)
Second time	4 (19)
Fifth time	1 (4.8)
Possible relative etiological factor	
Catching colds	6 (28.6)
Alcoholism	1 (4.8)
Treatment	
Diuretic	8 (38.1)
Glucocorticoid	5 (23.8)
Diuretic and glucocorticoid	6 (28.6)
ACEI	4 (19.0)

Days between arrival and hospitalization: 2.9±1.87. WBC, white blood cell; HACE, high-altitude cerebral edema; ACEI, angiotensin-converting enzyme inhibitor; ECG, electrocardiogram; HAPE, high-altitude pulmonary edema; SO_2_, oxygen saturation.

**Table III:** Comparison among HAPE patients and two control groups.

Group	n	NT-proBNP (pg/ml)^1^	EPO (mU/ml)^2^	VEGF (pg/ml)^3^	NO (*μ*mol/l)^4^
HAPE patients^a^	21	110.33 (87.18, 147.84)	59.25 (32.25, 327.00)	108.21 (85.35, 129.68)	25.00 (20.00, 50.00)
High-altitude control^b^	12	99.80 (85.47, 109.01)	67.40 (48.25, 324.35)	100.20 (74.66, 116.70)	55.43 (46.77, 67.04)
Plain controls^c^	13	72.02 (57.83, 94.76)	25.66 (10.65, 32.86)	60.51 (46.54, 70.53)	25.00 (12.50, 50.00)
P-value		0.010	0.001	0.002	0.013

Results are medians (25th, 75th percentiles).

1: a vs. b, P=0.309; a vs. c, P=0.002; b vs. c, P=0.019.

2: a vs. b, P=0.637; a vs. c, P=0.002; b vs. c, P=0.001.

3: a vs. b, P=0.308; a vs. c, P=0.007; b vs. c, P=0.001.

4: a vs. b, P=0.005; a vs. c, P=0.559; b vs. c, P=0.030.

HAPE, high-altitude pulmonary edema; EPO, erythropoietin; VEGF, vascular endothelial growth factor; NT-proBNP, N-terminal probrain natriuretic peptide.

**Table IV:** Comparison of HAPE patients pre- and post-treatment.

Period	n	NT-proBNP (pg/ml)	EPO (mU/ml)	VEGF (pg/ml)	NO (*μ*mol/l)
Pre-treatment	5	110.33 (93.46, 545.37)	182.52 (71.67, 348.49)	204.52 (74.87, 222.55)	80.13 (59.36, 134.56)
Post-treatment	5	56.95 (48.86, 91.59)	43.48 (26.25, 56.68)	196.52 (94.35, 243.65)	98.16 (85.06, 108.91)
P-value		0.043	0.043	0.500	0.686

Results are medians (25th, 75th percentiles). HAPE, high-altitude pulmonary edema; EPO, erythropoietin; VEGF, vascular endothelial growth factor; NT-proBNP, N-terminal probrain natriuretic peptide.
